# Research progress on risk factors of postoperative functional defecation disorder in anorectal malformation

**DOI:** 10.1002/pdi3.24

**Published:** 2023-08-30

**Authors:** Chenzhu Xiang, Xiao Xiang, Wei Feng, Yi Wang

**Affiliations:** ^1^ Department of General and Neonatal Surgery Children's Hospital of Chongqing Medical University National Clinical Research Center for Child Health and Disorders Ministry of Education Key Laboratory of Child Development and Disorders Chongqing Key Laboratory of Pediatrics Chongqing China

**Keywords:** anorectal malformation, functional defecation disorder, review, risk factors

## Abstract

Anorectal malformation (ARM) is a common congenital gastrointestinal malformation in neonates, which has complex pathological changes and unclear etiology. The only treatment modality currently available for ARMs is through surgical treatment to correct the deformity of the anal morphology and restore normal defecation function. In addition, the mortality rates of children with ARMs have significantly decreased as a result of recent advances in medical technology. It is important to note that despite the treatment of perianal anatomical deformities, approximately one‐third of ARM patients continue to experience functional defecation disorder (FDD), which includes fecal incontinence and constipation and negatively impacts the patient's quality of life. There are still numerous questions regarding the exact cause of FDD in ARM patients following surgery. It is generally accepted that the occurrence of FDD may be related to clinical staging, structural changes in the perianal rectal muscles, anomalies of the enteric nervous system, surgical injury, etc. However, some of these factors are still controversial and require additional study to be confirmed. In this paper, we review the risk factors for FDD following surgery in ARMs, which will help us to subsequently develop appropriate interventions to enhance the anal function and improve quality of life of children with ARMs.

## 1 INTRODUCTION

Anorectal malformation (ARM) is a common congenital problem and the incidence of ARMs is about 1 in 5000^1^ and more common in male. ARMs have unclear etiology, complex pathology, a wide variety of subtypes, and are often associated with other systemic malformations. If the patient is not treated in time, their life may be in danger. Currently, there are two main surgical treatments, namely laparoscopic‐assisted anorectoplasty (LAARP) and posterior sagittal anorectoplasty (PSARP). In recent years, surgical techniques and perioperative management have been continuously improved. However, most patients still face varying degrees of postoperative functional defecation disorder (FDD) in the short or long term.^2^, ^3^, ^4^ FDD includes irregular defecation movements (e.g., constipation and fecal incontinence) and uncontrolled bowel movements (e.g., soiled feces). These disorders not only affect the mental and physical health of the patients but also add to the burden of their family and society. Therefore, for improving their defecation function and living quality, it is particularly important to investigate the risk factors and develop appropriate interventions. The author describes the risk factors for postoperative FDD, which involve very complicated pathological changes and regulatory processes.

## 2 PHYSIOLOGICAL PROCESS OF DEFECATION

Bowel movement is a fundamental physiological process, leading to the excretion of feces, and is governed by a series of complicated and coordinated physiological reflex activities in the human body. Normal bowel function is due to a variety of factors, such as morphologically intact gastrointestinal tract, the coordinated interplay between muscle contractions and neuronal impulses.^5^

Defecation is performed in the following ways: firstly, the contraction of colon can help mix the intraluminal contents, soak up water, and pushing contents to the rectum. The entry of feces into the rectum can cause reflexive relaxation of the internal anal sphincter (recto‐anal inhibitory reflex, RAIR). When RAIR occurs, fecal matter moves further into the anal canal, which arises the feeling of needing to defecate. At this moment, the brain sends out neural models that allow the active puborectalis muscles and external anal sphincter to relax, thus allowing for bowel movement.

When bowel movements are not permitted, people need to control voluntarily. Fecal continence is defined as voluntary bowel motions with no soiling. Continence is achieved by three main factors: (1) voluntary muscle structures; (2) anorectal sensation; (3) rectosigmoid motility.^1^, ^6^

The voluntary control of defecation is a physiological process. There are a lot of factors involved here: the strength of internal and external sphincter, the movement of pelvic floor muscles, reflex mechanisms, compliance regulation and rectal sensation, etc. Abnormalities in any one of them can lead to defecation dysfunction.

## 3 RISK FACTORS OF POSTOPERATIVE FDD

### 3.1 Congenital developmental defect

#### 3.1.1 Dysplasia of the perianal rectal muscles

It is well known that the normal peri‐anorectal muscle structure plays a decisive role in defecation control. In 1982, Pena^7^ highlighted the importance of the striated muscle complex for bowel control, which consists of the levator ani, the external anal sphincter, and the longitudinal striated muscle fibers. The levator ani (composed of the iliococcygeus, puborectalis, and pubococcygeus) can support the pelvic organs and ensure the excretory function through coordinated contraction and relaxation.^8^ The external anal sphincter may be responsible for generating the maximum squeeze pressure in the anal canal, which involves in the acute control of stool.^9^ In addition, the internal anal sphincter is also involved in bowel control and dysplasia of the internal anal sphincter can weaken the recto anal inhibitory reflex and reduce the resting anal pressure, ultimately leading to constipation.^10^

Patients with ARMs especially the intermediate‐to high‐type malformation often show the dysplasia of perianal anorectal muscles, and the higher malformation, the more severe consequences. When these peri‐anorectal muscles, which are involved in the bowel movement, are poorly developed, peristalsis disorder occurs in the intestine, leading to fecal incontinence, constipation, and other dysfunctions, which seriously affects the prognosis of the patient.

Most of these conclusions are reasonable conjectures based on pathophysiological theories. However, the mechanism of interaction between abnormal perianal muscle development and postoperative defecation dysfunction is not clear. There is a lack of objective standards for the growth of pelvic floor muscles in children at present. Therefore, a standardized and multicenter randomized controlled study needs to be validated.

#### 3.1.2 Anomalies of the enteric nervous system

The enteric nervous system (ENS) is a reticular system composed of neurons, neurotransmitters, proteins, and supporting cells embedded within the gut wall. The ENS extensively contains the submucous plexus, which takes part in gastrointestinal secretion and absorption, and the intermuscular plexus, which takes part in the propulsion of the contents.^11^, ^12^ The maldevelopment of the plexus and/or the loss of the neurons leads to disturbed gastrointestinal function.

Patients with ARMs often have varying degrees of ENS abnormalities. Meier‐Ruge^13^ has reported that the incidence of ENS abnormalities in the distal rectum in patients with anal atresia was as high as 60%, mainly containing aganglionosis and neuronal dysplasia. Some scholars reported that abnormal ENS may lead to postoperative organ constipation and incontinence, for example, dysplasia of myenteric plexus and decrease of Cajal mesenchymal cells.^13^, ^14^ The most common abnormalities of ENS are intestinal anganglia and intestinal plexus dysplasia. Holschneider^15^ also found that the above two changes occurred in the distal rectum intestinal wall and believed that it was related to postoperative constipation. Mauricio et al.^16^ confirmed through animal experiments that the above changes in the terminal bowel of rats with ARMs can directly affect the anal function. Therefore, anomalies of the ENS may be one of the risk factors for postoperative FDD.

#### 3.1.3 Associated anomalies of other organ systems

Children with ARMs often have anomalies of one or more other organ systems with an incidence of approximately 40%–70%.^17^, ^18^ These associated malformations can be grouped into the VACTERL association (Vertebral, Anal, Cardiac, Tracheo‐Esophageal, Renal, and Limb). Of these, structural malformations of the urinary tract, spine/spinal cord, and heart are more common.^19^ Patients who have other systemic malformations tend to face more complex conditions. This means that these are more difficult to operate and the prognosis is relatively poorer. The previous retrospective study concluded that patients who have other systemic malformations had poorer postoperative defecation functions than those without other malformations.^20^

#### 3.1.4 Associated spinal malformations

For ARM patients with spinal malformation, their postoperative urination and defecation are poor.^19^, ^21^ Patients with more than two missing sacrums or other significant sacral deformities (such as hemivertebrae and fused vertebrae) have a worse item of outcomes than those with normal sacrums or lesser degrees of sacral dysplasia.^22^ The sacral ratio, introduced in 1995 and can be simply measured on a sacrococcygeal X‐ray, is now often used as a quantifiable measure of sacral development and partly reflects the degree of sacral development and the deficiency in spinal innervation. Previous study by Hendrik et al.^19^ has shown that a low sacral ratio is indeed a risk factor for postoperative FDD. However, there is a wide range of normal values for sacral ratio. The values may be influenced age and other factors, which may lead to certain errors.

#### 3.1.5 Associated spinal cord abnormalizes

There are several possible mechanisms of how spinal cord abnormalities lead to postoperative FDD. (1) The low‐level center for defecation is located in the lumbosacral spinal cord. When abnormalities occur in the sacral medulla and sacral nerves, it may affect the control of the defecation and therefore postoperative FDD occurs. (2) The medial part of the anterior pedicle of the sacral pulp acts is the motor center that controls the pelvic floor muscles and the external anal sphincter, which means that the abnormal sacral medulla can also affect the normal contractile function of the rectum and sphincter. (3) Lesions of the spinal cones, such as spinal cord embolism, could be a risk factor for postoperative FDD, because the spinal cones are also implicated in the regulation of defecation. (4) In addition, pre‐sacral spinal bulges may also cause constipation by directly compressing the rectum.

However, some studies have concluded that the spinal/spinal cord malformations and other related malformations have no significant effect on intestinal functions.^3^, ^23^ Therefore, larger sample sizes or further studies are needed to determine the specific effects between other systemic malformations and bowel functions.

### 3.2 Pathological classification

**FIGURE 1:**
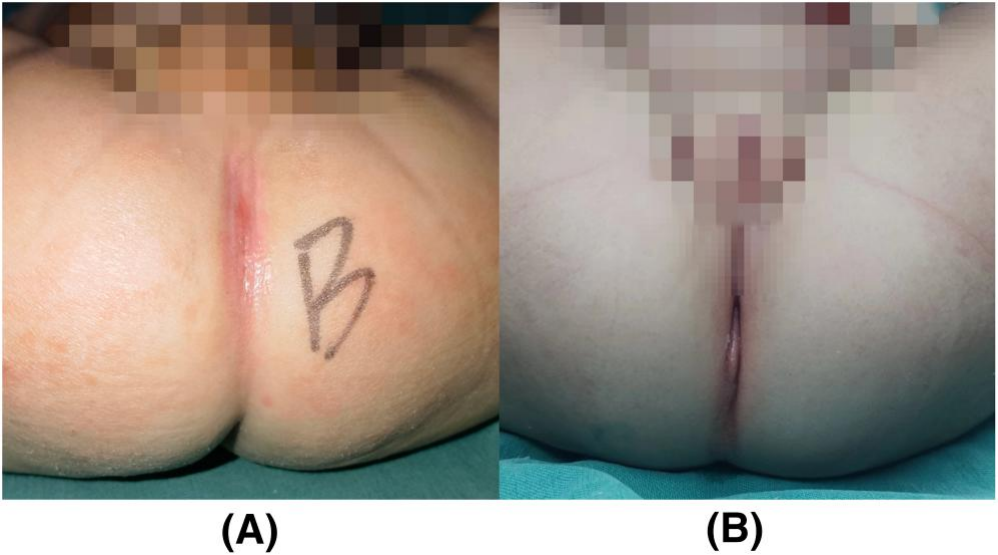
(A) Recto‐urethral prostate fistula, (B) cloaca anomaly.

The current common classification standard for ARM is the 2005 Krickenbeck classification standard, which is more widely accepted and used than the 1994 Wingspread classification standard. Because the Krickenbeck classification standard is based on precise anatomical abnormalities, it is more useful in guiding surgical approaches and predicting clinical outcomes. There are five classifications in male patients: recto‐perineal fistula, recto‐urethral bulb fistula, anal atresia without fistula, recto‐urethral prostate fistula, and recto‐vesical neck fistula (Figure 1A). Female anomalies have five classifications: recto‐perineal fistula, recto‐vestibular fistula, anal atresia without fistula, recto‐vaginal fistula, and cloaca anomaly (Figure 1B).

The pathological classification is considered to be one of the hazard factors for postoperative FDD in ARMs. The location and degree of occurrence of different classifications of ARM malformations mean that the development of the perianal muscles and nervous system varies, ultimately leading to differences in postoperative defecation functions.

By analyzing the clinical characteristics of 44 children with ARMs, Peter et al. found that postoperative fecal continence varied between patients with different ARM classifications. The self‐control rate of patients with recto‐perineal fistula was the highest and that of patients with cloacal ectopia was the lowest.^23^ This is the same as most of the previous studies that the more complex and higher location ARM, the poorer postoperative bowel movement autonomy.^24^, ^25^, ^26^, ^27^

For low‐type ARMs (recto‐perineal fistula and recto‐vestibular fistula), the patients often have more severe constipation. Although there is relatively little intraoperative dissection of the surrounding tissue, the prognosis for defecation control function is theoretically better.^28^, ^29^ Currently, the exact mechanism of constipation is not fully clear, which may be related to the following factors: intestinal motility disorders and abnormalities in the ENS.

In conclusion, constipation, the most common functional disorder in patients with ARMs, most often occurs in the low types with better positioned malformation.^30^ On the contrary, the incidence of constipation was lower in patients with higher malposition and that of fecal incontinence was higher due to poor bowel control.

### 3.3 Surgery operation

#### 3.3.1 Surgical approach and preoperative decisions

Posterior sagittal approach to sacral perineal anoplasty (PSARP), first reported by Pena^7^ et al. in 1982, and laparoscopic‐assisted anorectoplasty (LAARP), first reported by Georgeson^31^ et al. in 2000, are currently the two most common surgical approaches for moderate‐to‐high type ARMs. The effects of PSARP and LAARP on postoperative bowel function are still controversial.^32^, ^33^ At present, there is no proof of discrepancy in defecation function between the PSARP and LAARP.^34^, ^35^, ^36^ A midterm follow‐up study of moderate‐type ARMs showed no discernible difference in postoperative bowel function between patients receiving LAARP and patients receiving PSARP.^37^ It is worth noting that the mean age at the assessment of the LAARP group was significantly younger than that of the PSARP group in this study. And it is well known that these patients with ARMs tend to establish bowel habits and improve their defecation function as they get older with the administration and training.^24^ As a result, it is anticipated that the LAARP group will experience an improved long‐term bowel function than the PSARP group afterward. In another meta‐analysis of patients with moderate‐to‐high type ARMs, the LAARP group also had fewer wound infections and dehiscence, higher postoperative anal resting pressure, and a lower incidence of grade 2 or 3 constipation compared to the PSARP group.^38^ Therefore, it is possible for LARPP to reduce the risk of neural and muscular injury involved in defecation and achieve bowel function that is at least as good as or possibly better than PSARP. Nevertheless, systematic, multicenter, prospective, randomized controlled trials are still needed to confirm this.

However, the decision to use LAARP surgical treatment for moderate ARMs (e.g., recto‐urethral bulb fistula, recto‐vestibular fistula, recto‐perineal fistula and anal atresia without fistula) implies the need for more anatomically loose operations, which may damage the pelvic nerve tubes and are susceptible to complications and FDD. Therefore, it is often more important to make the appropriate surgical decision for each case.

In addition, colostomy is an acceptable alternative in cases of high‐type ARMs and complicated conditions, where the defecation function is performed through a fistula instead of the original perineal anus. In the long term, it is unfavorable to the child's integration into the society. Therefore, it is often used as a delaying tactic, waiting until conditions allow for anoplasty to be performed in due course. The impact of staging colostomy on the prognosis of postoperative defecation function is not clearly demonstrated by the available data. There were also no discernible differences between single‐stage and staging operations in terms of the functional outcome of defecation in a recent meta‐analysis.^39^

In clinical practice, it has been discovered that some previously common surgical techniques, such as ventral perineal anoplasty, have poor surgery field exposure, more postoperative complications, and worse postoperative bowel function. Therefore, they are already rarely used clinically.

Perineal anoplasty is commonly used for low‐type ARMs, after which children often achieve fairly satisfactory bowel function. However, it should be noted that low‐grade ARMs have a better development of the perianal muscles and nervous system and a more favorable prognosis for bowel function compared to the medium‐type and high‐type ARMs and therefore will not be discussed or compared too much.

#### 3.3.2 Intraoperative injuries and number of operations

Correct surgical practice is one of the keys to preventing the FDD. If the pediatric surgeon performs the procedure incorrectly, the anus opening may be made too large or too small, the end of the rectum may not pass through the puborectalis muscle ring, and the sacral nerve and anal sphincter may be injured, which may require additional surgery to repair these damages after the first procedure.

The number of anoplasties is also thought to be another element of influence in postoperative bowel function. Rintala^24^ et al. showed that secondary surgery with a failed or inadequate first reconstruction had a poorer bowel functional outcome than a successful first repair. This may be due to that multiple operations lead to increased damage to the anal sphincter and peripheral nerves and other important vital tissues, thus affecting the ability to perceive feces, making it unable to control defecation, resulting in FDD. Ardelean^15^ et al. also reported that of the 41 children with ARMs treated with secondary surgery, 18 children were still able to control their bowel movements, while the rest required medication to maintain cleanliness of their underwear.

Additionally, postoperative defecation function is associated with short‐term complications (e.g., anal scar stenosis, rectal stenosis, and postoperative wound infection). Whether to conduct bowel management (e.g., biofeedback training) also has a significant impact on patients with ARMs.

## 4 SUMMARY

In conclusion, there are a number of risk factors for postoperative FDD in patients with ARMs: congenital dysplasia, clinical staging, surgical approach, surgical operation, intraoperative injuries, and the number of anoplasty.

However, the mechanisms of these factors are still unclear and some factors are still debated. There is frequently some interactions between different factors. Future studies should explore more influencing factors through standardized, multicenter, prospective randomized controlled studies. Studies should try to more clearly elucidate the mechanisms of its occurrence at different levels, such as molecular biology and histoembryology.

Currently for pediatric surgeons, in order to prevent FDD in ARM patients, it is important to improve knowledge of the anatomy of the peri‐anorectal muscle groups, to choose the appropriate surgical approaches, and to avoid intraoperative injury. It is essential for patients with suspected malformations to identify and treat early. In addition, early bowel management should be emphasized. Adjusting intestinal function and establishing a good bowel pattern through a series of training can help treat FDD.

## AUTHOR CONTRIBUTIONS

Chenzhu Xiang is responsible for writing the first draft and translating. Xiao Xiang is responsible for translating and revising. Wei Feng is responsible for revising. Yi Wang is responsible for reviewing and revising.

## CONFLICT OF INTEREST STATEMENT

The authors have no conflicts of interest or financial ties to disclose.

## ETHICS STATEMENT

Not applicable.

## PATIENT CONSENT STATEMENT

All the patients' parents or legal guardians provided informed consent for using their medical records.

## Data Availability

Data sharing is not applicable to this article as no datasets were generated or analyzed during the current study.
